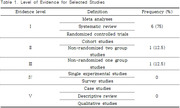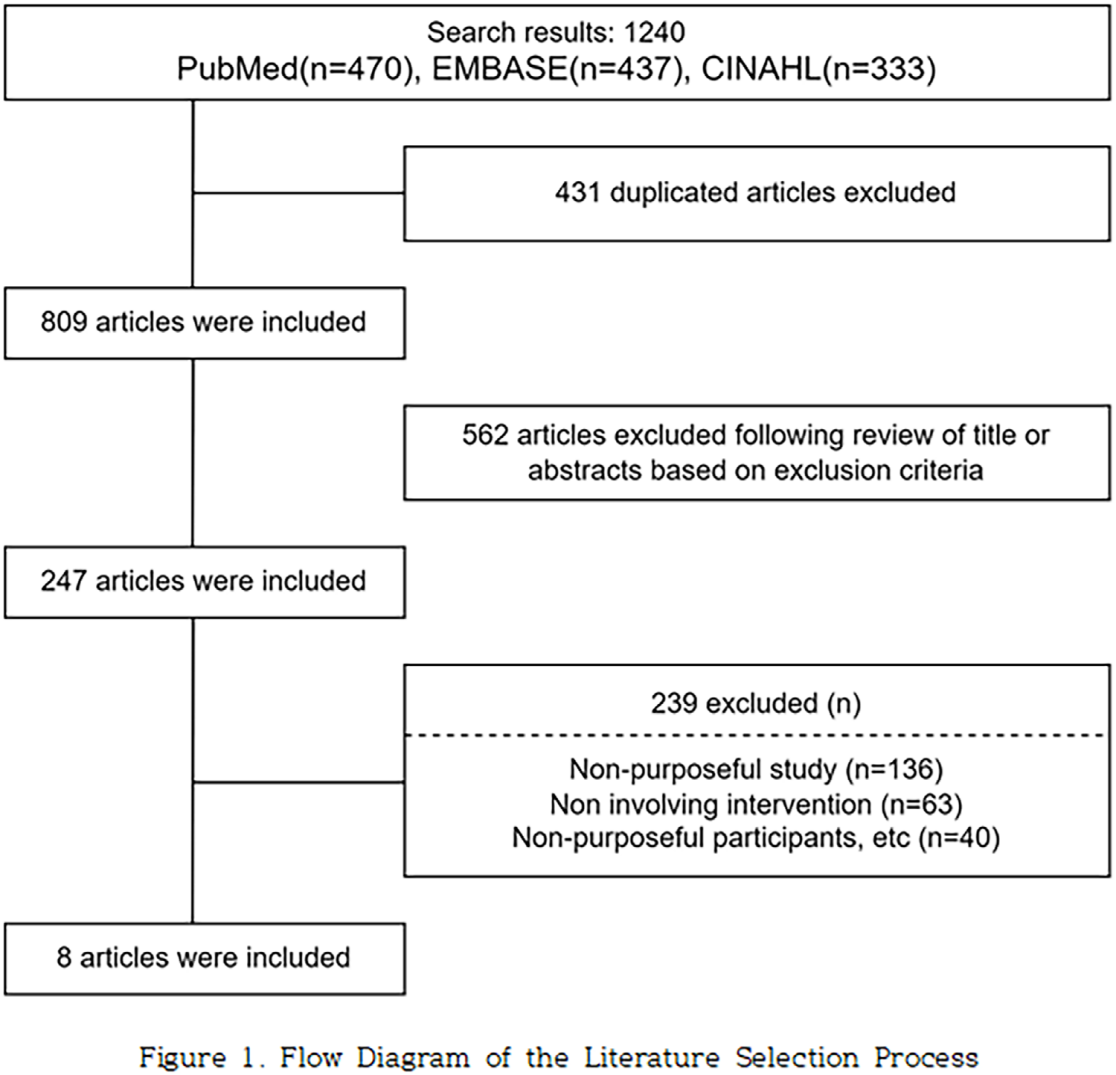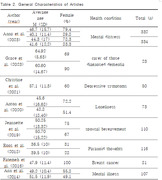# A Systematic Review of Interventions Targeting Social Isolation Among Middle‐Aged Adults

**DOI:** 10.1002/alz.094656

**Published:** 2025-01-09

**Authors:** Jiwon Shin, Hyun Yang, Sohyeon Yun, Inhye Kim, Hyunseo An, Hae Yean Park

**Affiliations:** ^1^ Graduate School, Yonsei University, Wonju, Wonju Korea, Republic of (South); ^2^ College of Software and Digital Healthcare Convergence, Yonsei University, Wonju, Heungup‐meon Korea, Republic of (South)

## Abstract

**Background:**

This systematic review aims to examine recent interventions targeting social isolation among middle‐aged adults, identifying trends in intervention types and their effectiveness. It highlights the urgent need for establishing a foundation for social isolation research across all ages.

**Method:**

A comprehensive literature search was conducted using PubMed, EMBASE, and CINAHL databases from January 1, 2014, to January 1, 2024. The search focused on terms related to social isolation and intervention programs. Eight studies were included, primarily randomized controlled trials.

**Result:**

The majority of interventions employed internet‐based methods, reflecting an adaptation to digital health trends. Interventions significantly reduced social isolation, particularly among female participants, and utilized various formats such as online interactions and group discussions. Notably, the use of the UCLA Loneliness Scale, Version 3 (UCLA‐LS‐3) was prevalent for measuring social isolation.

**Conclusion:**

The findings confirm the necessity for ongoing research into diverse and effective social isolation interventions. They serve as crucial evidence supporting the development of future interventions aimed at different age groups to combat social isolation effectively.